# Heterogeneity and plasticity of porcine alveolar macrophage and pulmonary interstitial macrophage isolated from healthy pigs *in vitro*

**DOI:** 10.1242/bio.046342

**Published:** 2019-10-15

**Authors:** Huan Liu, Jia Liu, Jing Huang, Xianchang Bai, Qinfu Wang

**Affiliations:** 1 College of Life Science and Technology, Dalian University, Dalian 116622, China; 2 Dalian Modern Agricultural Production Development Service Center, Dalian 116037, China

**Keywords:** Porcine alveolar macrophages, Pulmonary interstitial macrophages, Heterogeneity, Plasticity

## Abstract

This study investigated the heterogeneity and plasticity of porcine alveolar macrophages (PAM) and pulmonary interstitial macrophages (IM) isolated from healthy pigs, including phenotype, function and gene expression. Dynamic changes of nitric oxide (NO) levels secreted by PAM and IM with stimulation of different doses of lipopolysaccharide (LPS) were investigated by Griess method, and the viability of the PAM and IM cells was investigated by MTT assay. Flow cytometry, fluorescence quantitative PCR and ELISA techniques were used to measure cell phenotype, gene expression and cytokine secretion, respectively. The PAM and IM cells in normal healthy pigs showed heterogeneity with 95.42±1.51% and 31.99±5.84% of CD163+ macrophage, respectively. The NO level in IM was significantly higher versus PAM after LPS treatment. Consistently, the ratio of Arg I/iNOS in IM was much lower than that in PAM, suggesting that the PAM belong to M2 macrophages and the IM belong to M1 macrophages. The PAM and IM cells in normal healthy pigs also showed plasticity. The Arg I/iNOS ratio and TIMP1/MMP12 ratio were significantly decreased in LPS- or LPS+IFNγ-treated PAM and IM, suggesting that cells were polarized towards M1 macrophages under LPS or LPS+IFNγ stimulation. On the contrary, IL-4 and IL-13 stimulation on PAM and IM lead to M2 polarization. A similar result was found in IL-1β gene expression and TNFα secretion. In conclusion, porcine macrophages have shown heterogeneity and plasticity on polarization under the stimulation of LPS, IFNγ, IL-4 and IL-13.

## Abstract (precis)

**Summary:** The heterogeneity and plasticity of macrophages will be helpful for the understanding of pathologic mechanisms and the prevention of swine infectious disease.

## INTRODUCTION

Macrophages form a front line of the host defense system that can express diverse activities depending on the stimuli encountered. Many of these activities appear to be mutually antagonistic in nature, such as pro-inflammatory versus anti-inflammatory. To determine the macrophage functional status, readouts such as cytokine expression patterns and the ratio of arginase I (Arg I) to inducible nitric oxide synthase (iNOS) or the ratio of the tissue inhibitor of Metalloproteinases-1 (TIMP1) to the matrix metalloproteinases-12(MMP12) gene expression were used. What needs to be emphasized is that these functional phenotypes are reversible since macrophages are very flexible in adapting to changes in their microenvironment, suggesting their potential to be adjusted for therapeutic benefit.

In microenvironments dominated by lipopolysaccharide (LPS) or IFNγ, macrophages undergo M1 polarization, which leads to the increase of microbicidal capacity and inflammatory injury. M2 polarization of macrophages can be induced by IL-4 or IL-13, which mediates tissue repair and immune escape of pathogens, and thus lead to persistent infection. The inflammatory monocytes rapidly migrate into alveolar airspaces after lung infection and are reported to be the main effectors of acute lung injury and infection-related mortality (Duan et al., 2012). The *in vivo* function of less mature forms of porcine monocytes, which are believed to play an important role as precursors of inflammatory macrophages in mice and humans, has not been reported and remains completely unknown in pigs (Ondrackova et al., 2010). The aim of this study was to discover the heterogeneity and plasticity of porcine alveolar macrophage (PAM) and pulmonary interstitial macrophage (IM) cells in normal healthy pigs *in vitro*, which will be helpful for the understanding of pathologic mechanisms and the prevention of swine infectious disease.

## RESULTS

### CD163 expression on PAM and IM cells

**Fig. 1.**
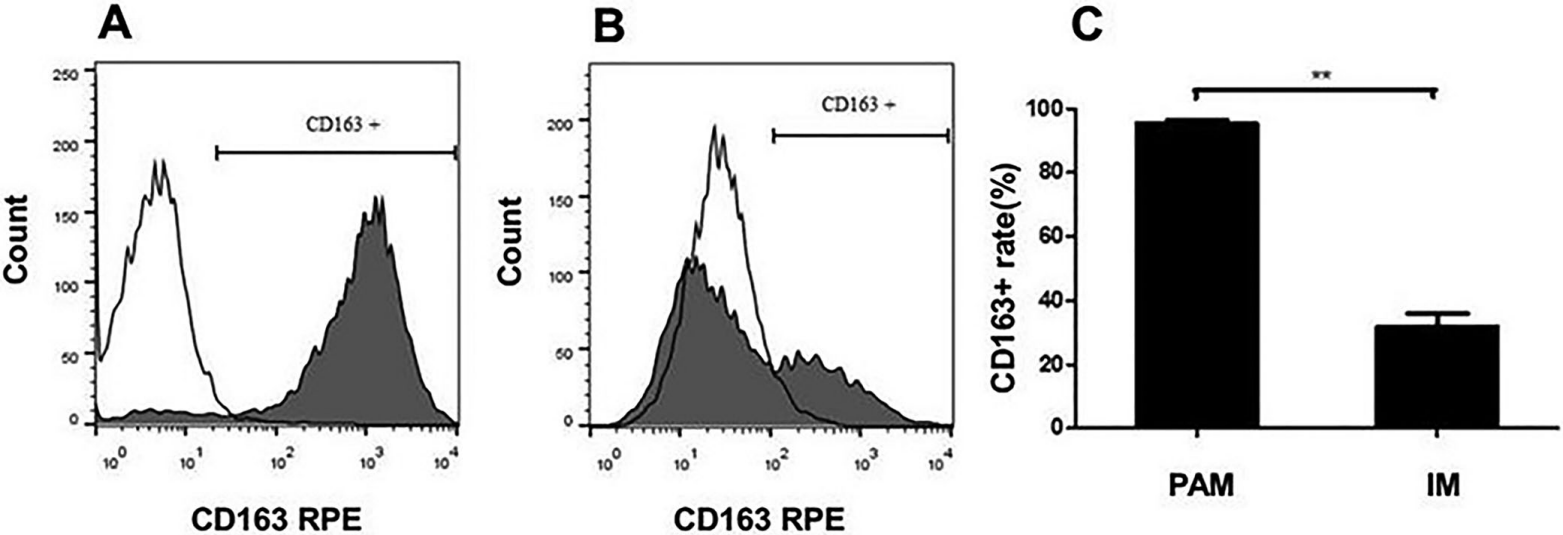
**CD163+ rates on PAM cells and IM cells isolated from healthy pigs.** Gray area represents the surface CD163 expression levels in PAM cells (A) and IM cells (B) and the white area represents the isotype control. **P<0.01.

The CD163 expression on PAM and IM cells isolated from healthy pigs was measured by Flow cytometry. The results showed that CD163+rate of IM cells was significantly decreased compared to that of PAM cells (*P*<0.01) (Fig. 1).

### Dynamic changes of nitric oxide (NO) level secreted by PAM and IM stimulated with different doses of LPS

**Fig. 2.**
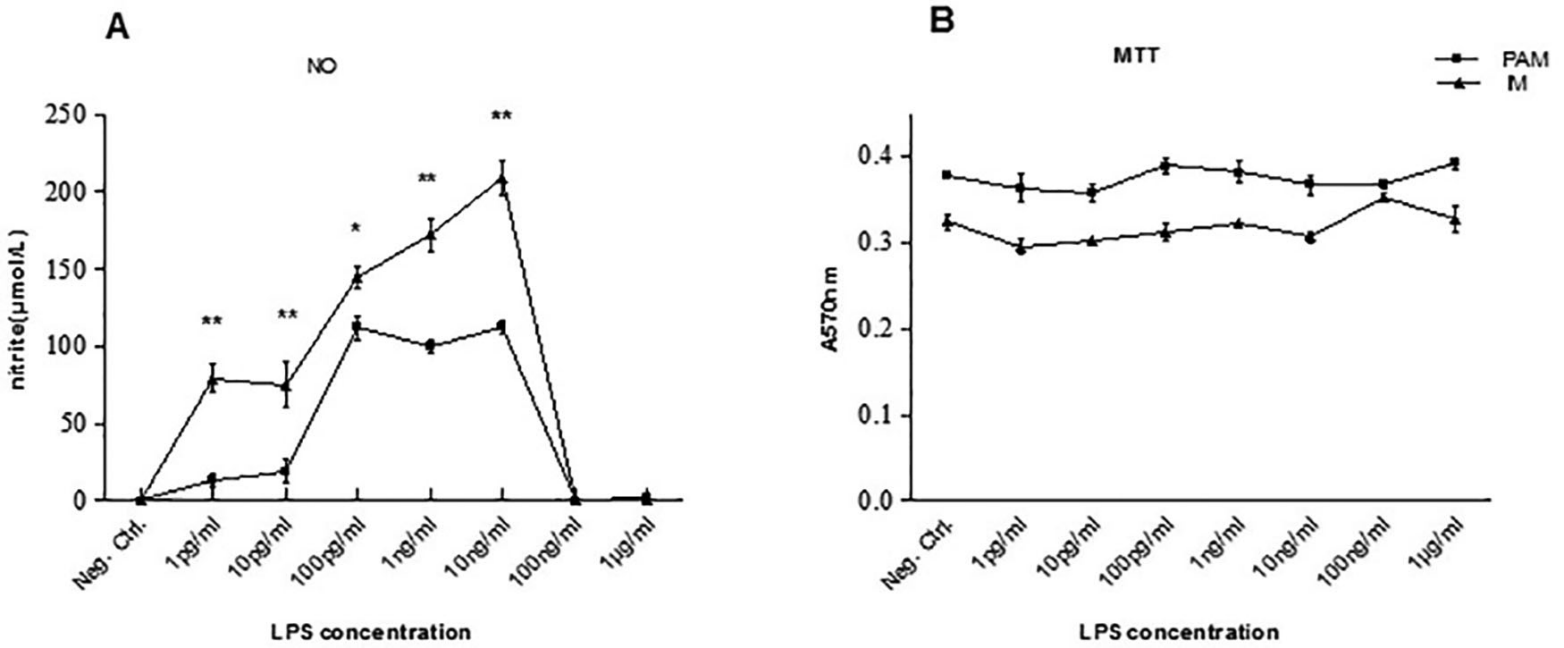
**Dynamic changes of NO levels in PAM and IM cells stimulated by different doses of LPS (A) and the viability of the PAM and IM cells (B) *in vitro*.** *P<0.05, **P<0.01.

PAM and IM cells isolated from healthy pigs were stimulated with different doses of LPS (1 pg/ml, 10 pg/ml, 100 pg/ml, 1 ng/ml, 10 ng/ml, 100 ng/ml and 1 μg/ml) *in vitro* and then NO level and cell viability were examined (Fig. 2).

With Griess method, we observed increased NO levels in the supernatant of PAM and IM cells treated with LPS in a dose-dependent manner, which showed a continued increase of NO levels from 1 pg/ml to 10 ng/ml and reached its peak at 10 ng/ml LPS treatment. However, the NO levels decreased sharply with higher doses of LPS treatments at 100 ng/ml and 1 μg/ml. The viability of the PAM and IM cells were assessed by MTT assay, and the result showed no significant changes among the groups treated with various doses of LPS.

### Arg I/iNOS ratio in PAM and IM cells treated with LPS

**Fig. 3.**
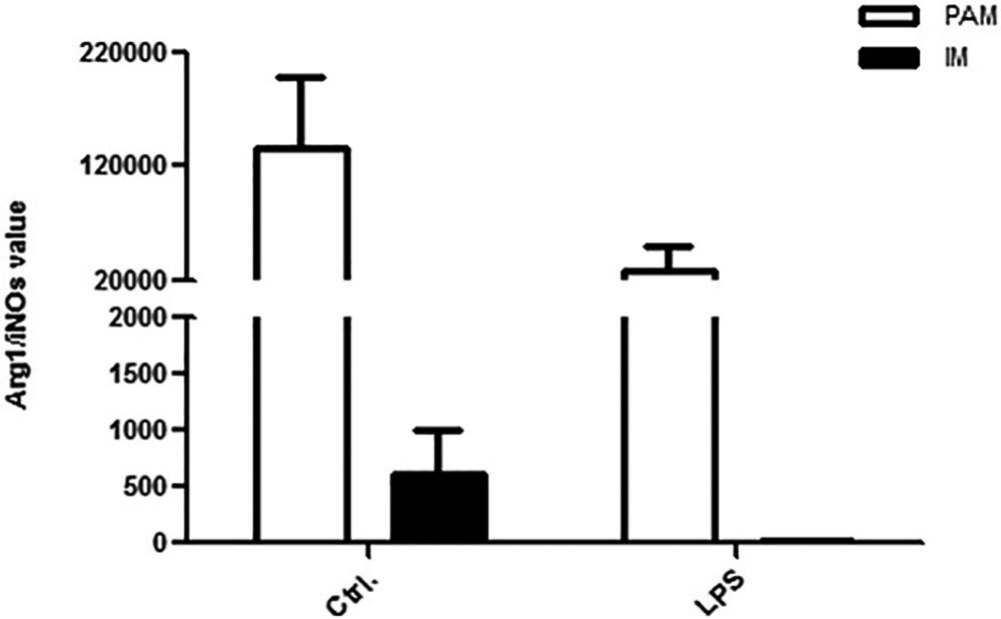
**Arg I/iNOS ratios in PAM and IM cells treated with LPS versus control.**

Arginase (Arg I) and iNOS are phenotype markers of differently polarized macrophage of pro-inflammatory cells (M1) and anti-inflammatory cells (M2), respectively. Arg I/iNOS ratio was calculated by dividing the relative amount of Arg I mRNA with the relative amount of iNOS mRNA in PAM and IM cells isolated from healthy pigs (Fig. 3).

The results showed that the Arg I/iNOS ratio in IM cells was significantly lower than that in PAM cells, indicating the macrophages from the lung of swine were differentially polarized. The IM cells were M1 cells, and the PAM cells were M2 cells. After treatment of LPS, the Arg I/iNOS ratios in both PAM and IM cells significantly decreased, which was consistent with the results of NO levels in PAM and IM cells.

### Plasticity of PAM cells and IM cells isolated from healthy pigs

**Fig. 4.**
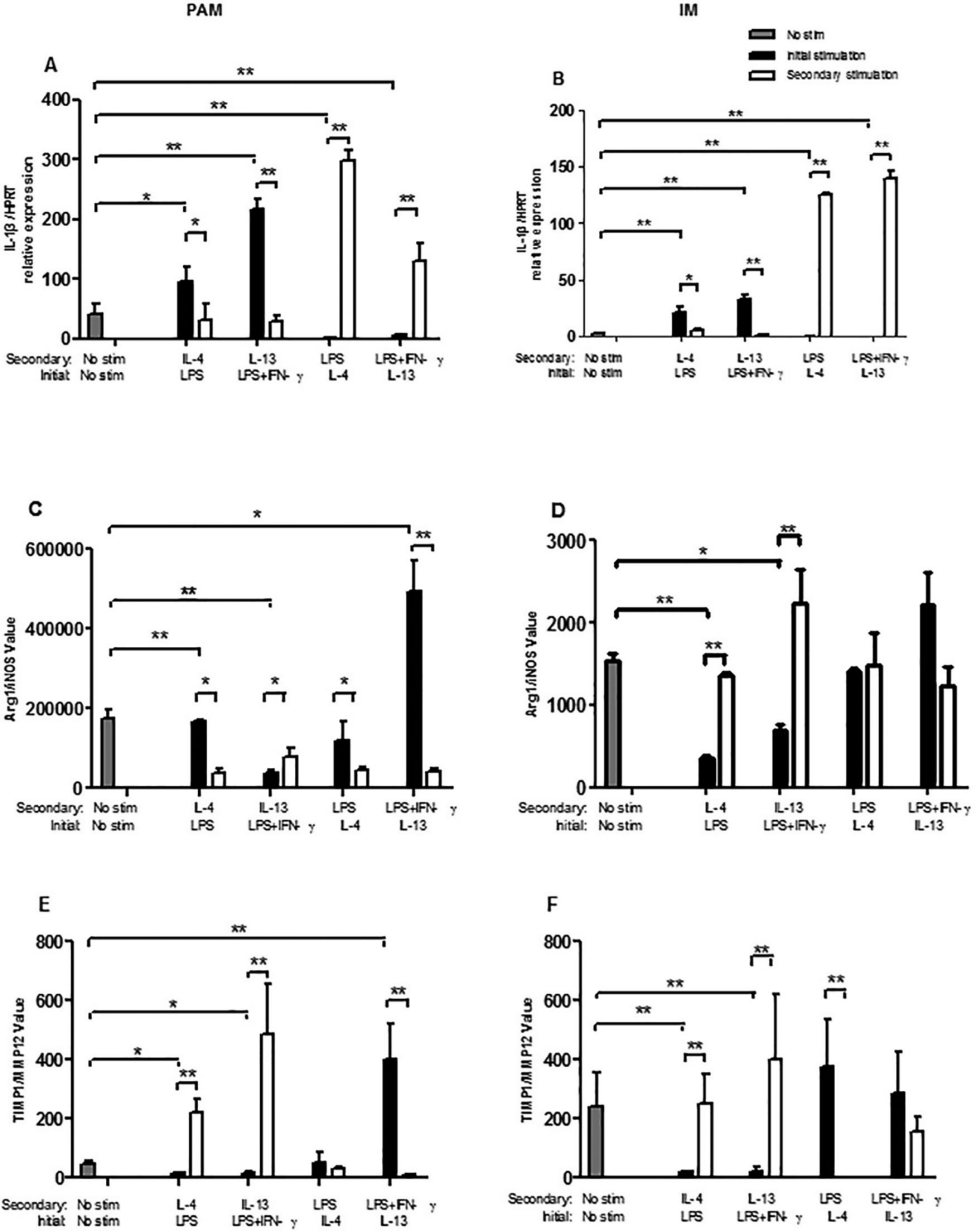
**Plasticity of PAM cells and IM cells isolated from healthy pigs after initial and secondary treatment *in vitro*.** Plasticity of PAM cells and IM cells isolated from healthy pigs was described by the IL-1β/HPRT relative expression (A,B), the Arg1/iNOS ratio (C,D), TIMP/MMP12 ratio (E,F). PAM cells were shown in the left panel (A,C,E) and IM cells were shown in the right panel (B,D,F). *P<0.05, **P<0.01.

Plasticity of PAM cells and IM cells isolated from healthy pigs was described by the IL-1β/HPRT relative expression (Fig. 4A,B) and the ratio of Arg1/iNOS (Fig. 4C,D) and TIMP1/MMP12 (Fig. 4E,F).

The PAM and IM cells were divided into five groups (A, B, C, D and E) and received two rounds of treatment. The initial treatment was achieved by adding 10 ng/ml of LPS (group A), 10 ng/ml of LPS plus 20 ng/ml IFNγ (group B), 25 ng/ml of IL-4 (group C), 50 ng/ml of IL-13 (group D), and medium control (group E). After 24 h, the cells received the secondary treatment by espousing to IL-4 (group A), IL-13 (group B), LPS (group C) and LPS+IFNγ (group D) for an additional 24 h. At the end of each round treatment, cells were harvested to extract total RNA and the relative expression of each gene indicated in the Fig. 4A to F was analyzed by qPCR.

As shown in Fig. 4A, the gene expression level of IL-1β was significantly increased in both PAM and IM cells treated with LPS or LPS+IFNγ in both rounds of treatment (*P*<0.01). In contrast, both rounds of treatment with IL-4 or IL-13 resulted in decreased levels of IL-1β transcript in both PAM and IM cells (*P*<0.01) (Fig. 4B). From the results in Fig. 4C, we observed that the initial treatment with LPS or LPS+IFNγ induced a lower ratio of Arg I/iNOS (*P*<0.01) compared with the control group, and the secondary treatment with LPS or LPS+IFNγ also induced a lower ratio of Arg I/iNOS compared with that before treatment (*P*<0.05 for LPS, *P*<0.01 for LPS+IFNγ). Whereas, PAM cells showed increased ratio of Arg I/iNOS after secondary treatment with IL-4 or IL-13 (*P*<0.05) and a similar result was found in PAM cells after initial treatment with IL-13, but not in IL-4 treatment. Similarly, the Arg I/iNOS ratio was significantly increased in IM cells after receiving secondary treatment with IL-4 or IL-13 (*P*<0.01), but not in initial treatment with IL-4 or IL-13 (Fig. 4D). We also examined the ratio of TIMP1/MMP12 as an additional marker for macrophage polarization (Fig. 4E,F). In alignment with the results of Arg I/iNOS ratio in both PAM and IM cells, TIMP1/MMP12 ratio also showed a significant increase after secondary treatment of IL-4 or IL-13 in both PAM and IM cells (*P*<0.01 for both cells). In contrast, a significant decrease of TIMP1/MMP12 ratio was recorded after the first round treatment with LPS or LPS+IFNγ in both PAM and IM cells (*P*<0.05 for PAM, *P*<0.01 for IM).

### TNFα production of PAM and IM cells in healthy pigs

**Fig. 5.**
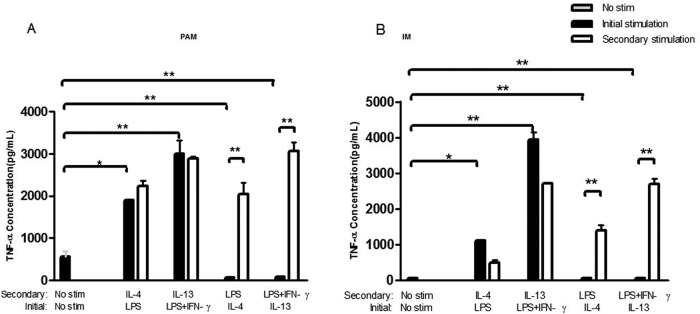
**TNFα production of PAM cells and IM cells isolated from healthy pigs after initial and secondary treatment *in vitro*.**

PAM and IM cells isolated from healthy pigs were treated with a two round procedure as outlined above, and at the end of each round of stimulation the TNFα amount in supernatant was assayed by ELISA (Fig. 5A,B).

The results showed in Fig. 5 revealed that the differential effect of TNFα secretion in macrophages treated with different cytokines. The TNFα levels was significantly increased in culture medium of both PAM and IM cells received treatment with LPS or LPS+IFNγ in both rounds of treatment (*P*<0.01). In contrast, decreased production of TNFα was found in PAM and IM cells in the presence of IL-4 or IL-13 in first round of the treatment (*P*<0.01).

## DISCUSSION

Both PAM cells and IM cells were previously reported to play critical roles in shaping the outcomes of lung inflammation after infection (Schneberger et al., 2012). The PAM and IM cells have different polarization, and are heterogeneous in morphology. The PAM cells are bigger in size and more granular than IM cells (Hu et al., 2016). The PAM and IM cells were reported to be able to polarize between classical M1 (CD80+) and M2 (CD163+) phenotypes in human and in mice (Edin et al., 2012; Sun et al., 2018). However, there is no well-characterized marker for porcine macrophage (Bullers et al., 2014). In healthy adult lungs, PAM cells exist as the predominant cell type (>98%) in the alveolar airspaces and nearly all alveolar macrophages in healthy pigs are of CD163+ phenotype (Jung et al., 2010). The phenotypes of CD163 expression in PAM and IM cells were detected with FCM analysis (Fig. 1). Results showed that porcine M1-IM expressed lower CD163 level and porcine M2-PAM expressed higher CD163 level.

NO production in mammalian macrophages is known to play a significant role in intracellular defense against a number of microbial infections. NO is an important early signal in many pathophysiologic processes. The results in Fig. 2 showed that 10 ng/ml LPS was the optimal dose for NO production in stimulating PAM and IM cells and the NO level was undetectable in 100 ng/ml and 1 μg/ml LPS groups. The viability of the PAM and IM cells were shown that no significant changes among these groups, which suggested that the sharp decrease in NO production at 100 ng/ml and 1 μg/ml LPS treated PAM and IM cells was not due to the harm of high doses of LPS to these cells. The NO level in the supernatant of IM cells treated with different doses of LPS was much higher than in PAM cells (*P*<0.05 or *P*<0.01), indicating that IM cells were more prone to be M1 cells, and the PAM cells were more prone to be M2 cells.

The production of NO is catalyzed by iNOS and Arg I acts on L-Arg, which is the same substrate that is acted upon by iNOS. This competition for substrate acts as an approach to control the production of NO (Nelson et al., 2011). The mRNA expression of Arg I and iNOS genes are marker genes for M2 and M1 polarization, respectively. Therefore, the levels of iNOS and Arg I are frequently used as indexes of the M1 and M2 functional macrophage phenotype, respectively (Chiang et al., 2008; Davis et al., 2013). Based our results in Fig. 3, the Arg I/iNOS ratio of IM was significantly lower than in PAM cells, indicating the heterogeneity in polarization of different macrophages in the lung of swine. Specifically, IM cells were M1 cells and the PAM cells were M2 cells. After treatment of LPS, the Arg I/iNOS ratio in PAM and IM cells significantly decreased, which was consistent with the results of NO levels in PAM and IM cells (Fig. 2).

From the results in Fig. 4, it can be concluded that PAM and IM macrophages exhibited plasticity. The initial stimulation of LPS, LPS+IFNγ on PAM and IM cells *in vitro* resulted in M1 polarization, while the stimulation of IL-4 and IL-13 resulted in M2 polarization. Furthermore, this initial polarization was completely reversed by the secondary stimulation using the opposite polarizing cytokines. LPS and IFNγ stimulated PAM cells are shown to express M1 molecules, whereas IL-4 and IL-13 stimulated PAM cells are shown to express M2 molecules (Wang et al., 2017). Macrophage arginase expression is upregulated in response to anti-inflammatory cytokines, such as IL-4 and IL-13, forming iNOS-expressing M1 cells into arginase-expressing M2 cells (Chen et al., 2014). The resulting Arg I/iNOS ratio in PAM cells by initial treatment with IL-4 and IL-13, showed that the IL-13 stimulated PAM cells expressed a higher Arg I/iNOS ratio versus IL-4 stimulation, suggesting that IL-13 is more critical in M2 polarization, compared to IL-4.

TIMP1/MMP12, another parameter of macrophage polarization, showed similar results as the Arg I/iNOS ratio in PAM and IM cells after the initial and secondary stimulation (Fig. 4E,F). The matrix metalloproteinases (MMPs) are zinc- and calcium-dependent enzymes that regulate the physiological and pathological metabolisms of collagen-based tissues (Cvikl et al., 2018). MMP12 is an elastase (also known as metalloelastase) that mainly functions in the degradation of elastin (Barroso et al., 2017). Like other members of MMP family, MMP12 is produced as a proenzyme, mainly by macrophages. TIMP-1 is a very potent inhibitor of MMPs, including MMP12 (Salmela et al., 2001). The skewing of macrophages to the M1 phenotype enhanced MMP expression and depressed TIMP expression, while skewing to the M2 phenotype enhanced TIMP1 expression (Annamalai et al., 2018). Therefore, the TIMP1/MMP12 ratio might be related to the polarization of macrophage, which indicates tissue remodeling (Bernasconi et al., 2015). The results in Fig. 4E and F suggested that the TIMP1/MMP12 ratio was relevant to the M2/M1 balance, showing plasticity in polarization of swine lung macrophages in the environment containing LPS, LPS+IFNγ, IL-4 or IL-13. MMP12 contributes to the proliferation of mouse macrophages as well as secretion of IL-1β, IL-6, TNFα through the ERK/P38 MAPK signaling pathway (Guan et al., 2019). The imbalance between TIMPs and MMPs has been implicated in the progression of inflammation, facilitating the understanding of pathologic mechanisms and prevention of swine infectious disease.

To further evaluate macrophage polarization plasticity, TNFα production levels of polarized PAM and IM cells after stimulation of LPS, LPS+IFNγ, IL-4 and IL-13 were analyzed by ELISA technique (Fig. 5). The TNFα production was significantly increased (*P*<0.01) in the initial or secondary LPS- or LPS+IFNγ-treated PAM and IM cells. On the contrary, the TNFα production was significantly decreased in initial IL-4- or IL-13-treated PAM cells and IM cells (*P*<0.01). All the results of IL-1β relative expression, the ratio of Arg I/iNOS, TIMP1/MMP12 and TNFα secretion in PAM and IM cells suggested that LPS and IFNγ stimulated PAM and IM cells polarized towards M1, and the IL-4 and IL-13 polarized towards M2. These data demonstrated that *in vitro* macrophages are capable of complete repolarization from M1 to M2 or M2 to M1, in response to changes in the cytokine environment. These changes in macrophage polarization are rapid and occur at the levels of gene expression, cytokine secretion and NO production. These findings not only revealed the dynamic changes in macrophage polarization, but also provided a basis for macrophage-centered diagnostic and therapeutic strategies (Sica and Mantovani, 2012; Zhu et al., 2014).

In summary, LPS, LPS+IFNγ, IL-4 and IL-13 stimulation differently induced M1 and M2 polarization, as indicated by the distinct expression of marker gene IL-1β mRNA, the ratio of Arg I/iNOS and TIMP1/MMP12, and TNFα protein production. Switching LPS to IL-4, and LPS+IFNγ to IL-13 stimulating condition, can result in uniform changes in profiles of polarization marker genes, and vice versa. Collectively, we can conclude that M1 and M2 macrophage polarization is highly plastic to the environment, and thus repolarizing macrophages could be beneficial to be included in the treatment of diseases.

## MATERIALS AND METHODS

### Ethics statement

The lung collection and the pig euthanization were approved by the Institutional Animal Care and Use Committee, Dalian University, China. The biological agents used in this study were handled as per the Institutional Biosafety Committee, Dalian University, China.

### Pigs and inoculations

This study was performed on a subset of Duroc-Landrace-Yorkshire crossbreed piglets, approximately 8–11 weeks of age and 9–12 kg in weight. They were commercially purchased from local areas in Dalian, China. The experimental pigs were seronegative for antibody to PRRSV at the beginning of the experiment by ELISA technique (Herd-Chek PRRS ELISA, IDEXX) and were also confirmed to be free of PRRSV, porcine circovirus type 2, pseudorabies virus, mycoplasma hyopneumoniae and classical swine fever virus in serum by PCR technique.

### Collection of porcine alveolar macrophage

Pigs were euthanized by exsanguination. The trachea was ligated to prevent total pulmonary collapse, followed by the removal of heart and lungs from the thorax. Alveolar macrophages were collected from lungs by bronchioalveolar lavage. The bronchial alveolar lavage fluid (BALF) was almost exclusively composed of PAM cells (Ait-Ali et al., 2007). The PAM cells were cultured in complete RPMI-1640 medium [10% fetal bovine serum (FBS), 2 mM L-glutamine, 1 µg/ml fungizone, 100 U/ml penicillin and 100 µg/ml streptomycin] in Petri dishes for 2 h at 37°C in a humidified 5% CO_2_ atmosphere. The non-adherent cells were removed by aspiration and the adherent cells were digested by trypsin, washed three times with complete RPMI 1640, and counted by Trypan Blue dye exclusion. The cells were plated in 96-well culture plates or six-well culture plates, at densities of 1×10^5^ cells/well or 1×10^6^ cells/well, respectively.

### Isolation of interstitial macrophages

The protocol of IM isolation was adapted from rat pulmonary interstitial macrophages preparation (Cong et al., 2002). To improve the purity of IM cells, pigs were euthanized by exsanguination and pulmonary vasculature was perfusated. Finally, the alveolar macrophages were collected from lungs followed by IM cells isolation. The lung tissue was chopped into pieces of less than 1 mm^3^ using scissors. To remove the remaining PAM cells and blood cells, the tissue was washed with PBS over a 100 μm cell strainer until the filtrate appeared to be clear. The tissue was then digested using 0.025% collagenase IA (Sigma-Aldrich, St Louis, MO, USA) for 60 min at 37°C in a shaking water bath. The digestion was filtrated by 200 mesh stainless steel filter, centrifuged at 1500 rpm for 10 min and re-suspended in RPMI 1640 culture medium with 10% FBS in Petri dishes. Following adherence to Petri dishes, the adherent cells were collected, washed, counted and recorded for viability and plated as PAM cells.

### NO assays

The PAM and IM cells were plated in triplicate at a density of 1×10^5^ cells/well in 96-well plates for 24 h and then stimulated with different doses of LPS at 1 pg/ml, 10 pg/ml, 100 pg/ml, 1 ng/ml, 10 ng/ml, 100 ng/ml and 1 μg/ml for 20 h. The culture media were collected for the NO assays. NO level was measured by the detection of its stable oxidative metabolite, nitrite. Briefly, 80 μl media were mixed with 80 μl Griess reagent (0.1% naphthylethylenediamine dihydrochloride and 1% sulfanilamide in 5% phosphoric acid) and then shaken for 5 min at room temperature. The nitrite concentration was measured by the absorbance at 550 nm and determined by a linear calibration curve (r^2^=0.9987) constructed with sodium nitrite standards as described by the equation of y=134.67x−0.2105.

### Cell viability assay

Cell viability was evaluated by MTT (3-[4,5-dimethylthiazol-2-yl]-2,5-diphenyl tetrazolium bromide) assay. The PAM and IM cells (1×10^5^/well) were plated in 96-well plates and treated with an increasing concentration gradient of LPS stimulation as in NO assay. After the media were removed, cells in each well were incubated with MTT in Phenol Red-free RPMI 1640 medium for 3.5 h according to the manufacturer's protocol. The assay was stopped by adding MTT solubilization solution (10% Triton X-100 and 0.1 N HCl in anhydrous isopropanol). The plates were stored overnight at 37°C to completely dissolve formazan crystals. Formazan was quantified by the absorbance at 570 nm using a Bio-Rad iMark microplate reader (Wang et al., 2009).

### Cytokine stimulation

The PAM and IM cells were seeded in triplicate into six-well plates and divided into five groups, A, B, C, D and E. Group A: 10 ng/ml of LPS (Bioengineering Shanghai Co., Ltd. China) added; group B: 10 ng/ml of LPS plus 20 ng/ml IFNγ (Biosource, USA) added; group C: 25 ng/ml IL-4 (BIOTECH, INC.) added; group D: 50 ng/ml of IL-13 (BIOTECH, INC.) added; and group E: medium control. After 24 h of treatment, the cells were espoused to fresh medium containing IL-4 (group A), IL-13 (group B), LPS (group C) and LPS+IFNγ (group D), respectively, for an additional 24 h. Then the supernatants were collected for enzyme-linked immunosorbent assay (ELISA) and cell pellets were processed to extract RNA and run qPCR analyses.

### RNA extraction and quantitative real-time polymerase chain reaction

Total RNA was prepared using Trizol reagent (Takara, China) based on the instruction by manufacture. Then, cDNA was synthesized by an oligo (dT)15 primer and SuperScript™ II reverse transcriptase reagents (Takara, China). Quantitative real-time PCR was performed with the iTaq™ Universal SYBR GREEN Supermix using a CFX96 Optics Module instrument (Bio-Rad, Hercules, CA, USA). The forward and reverse primer sequences were summarized in Table 1.

**Table 1.**
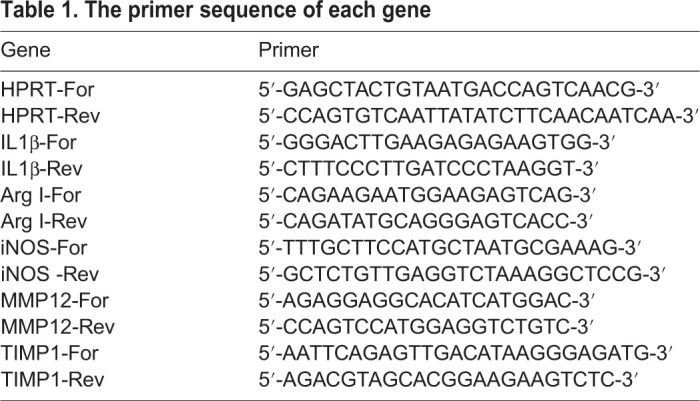
**The primer sequence of each gene**

The thermal cycling conditions were set as: 3 min at 95°C, followed by 35 cycles of denaturation at 95°C for 5 s, annealing at 55°C for 10 s, and extension at 72°C for 30 s. The fluorescence signal was detected at the end of each cycle. The results were analyzed by the CFX96 manager software supplied with the machine. A melting curve and electrophoresis were used to confirm the specificities of the products. The expression levels of each gene were normalized to the internal control HPRT.

### Flow cytometry analysis

Surface staining reagents including Mouse anti Pig CD163: RPE (MCA2311PE, Bio-Rad). After staining, cells were fixed in 1% formaldehyde and analyzed on FACScan (Becton-Dickinson, Franklin Lakes, NJ, USA) upgraded with a red laser (Cytek Development, Fremont, CA, USA). Data analysis was conducted using FlowJo software (Tree Star, Inc., Ashland, OR, USA).

### TNFα ELISA assay

The TNFα in the culture supernatants of PAM and IM treated by different cytokines were quantified using the porcine TNFα ELISA (R&D Systems, USA). The optical density A450 nm of each well was determined by Multi-function enzyme marker (Bio-Tek, USA).

### Statistical analysis

Each experiment was independently replicated on three individual pigs, and all experiments were replicated at least three times. Data were presented as mean±s.e.m. when indicated. Statistical analysis was conducted by *t*-test with a 95% confidence limit, and two-way ANOVA for multi-group comparisons by Prism 7.0 (GraphPad Software Inc., USA). Differences were considered significant at *P*<0.05. All data are representatives of at least three independent experiments.

### Compliance with ethical standards

Animals used in this study were euthanized as per the protocols approved by the Institutional Animal Care and Use Committee, Dalian University. The biological agents used in this study were handled as per the Institutional Biosafety Committee, Dalian University. The animal Ethics Committee approval number was DW2017-080.
